# Efficacy of Alpha Glucosidase Inhibitor from Marine Actinobacterium in the Control of Postprandial Hyperglycaemia in Streptozotocin (STZ) Induced Diabetic Male Albino Wister Rats

**Published:** 2018

**Authors:** S.R. Sathish Kumar, K.V. Bhaskara Rao

**Affiliations:** *Molecular and Microbiology Research Laboratory, Environmental Biotechnology Division, School of Bio Sciences and Technology, VIT University, Vellore - 632 014, Tamilnadu, India.*

**Keywords:** Streptomyces, Postprandial, α-glucosidase, Marine actinobacteria, Diabetes mellitus, Salterns

## Abstract

The current study was carried out to evaluate the *in-vitro* and *in-vivo* efficiency of alpha glucosidase inhibitor of marine actinobacteria in the control of postprandial hyperglycaemia. Soil samples were collected from salterns, coastal area in Kothapatnam, Ongole, Andhra Pradesh, India. Among the actinobacterial isolates tested for yeastα-glucosidase inhibitory activity, only three isolates showed prominent inhibition. The patient isolate was selected and identified as *Streptomyces coelicoflavus* SRBVIT13 using 16S r-RNA gene sequencing. In *in-vitro *studies, the chloroform extract of *Streptomyces coelicoflavus* SRBVIT13 showed significant enzyme inhibitory activity against yeast and mammalian α-glucosidaseenzymes. In animal studies, the oral ingestion of chloroform extract (600 mg/kg) of *S. coelicoflavus* SRBVIT13 in maltose and sucrose loaded diabetic rats, showed significant regulation of postprandial blood glucose by 82.25% and a 77.25% reduction, respectively. The lead compound from *S. coelicoflavus*SRBVIT13 was isolated, purified, characterized, and identified by stranded analytical techniques as 2-t-butyl-5-chloromethyl-3-methyl-4-oxoimidazolidine-1-carboxylic acid, t-butyl ester. The results obtained in the present study are promising and the bioactive compound from *S. coelicoflavus*SRBVIT13 may be considered as a potential agent in regulating the postprandial hyperglycaemia.

## Introduction

Diabetes mellitus is a chronic metabolic disorder characterized by the elevation of blood glucose level by the disturbances of carbohydrate, fat, and protein metabolism (1). Diabetes mainly occurs due to the insufficient secretion of insulin or insulin resistance which leads to elevation of blood glucose level (2). Diabetes mellitus is categorized into three types *Viz*., type I diabetes, type II diabetes, and Gestational diabetes. Type II diabetes is the fastest-growing metabolic disorder in many parts of the world. The incidence of type II diabetes in 2010 was estimated as ~171 million worldwide, and the numbers may be doubled by 2020 (3). Type II diabetes occurs due to impaired tolerance of glucose and leads to the formation of insulin resistance. Consequently, in type II diabetes β-cells fail to secrete the insulin hormone and affect the skeletal muscle, liver, and adipose tissues (4, 5). Furthermore, several factors, such as obesity, genetic alterations, hereditary, and other environmental factors, contribute major roles in the development of insulin resistance (6, 7).

Postprandial hyperglycemia (PPHG) is defined as a sudden increase in blood sugar level after a meal. In healthy persons, pancreas enhances the secretion of insulin to regulate the postprandial blood glucose level. In type II diabetes patients, the pancreas fails to secrete sufficient insulin or is insulin resistance which leads to postprandial hyperglycaemia (8). In this condition, after food intake, the production of insulin hormone is reduced and low secretion of glucagon leads to the improper metabolism of glucose in liver and kidney. Hence, there are no sufficient uptake of glucose and subsequently its blood levels increase (9, 10). PPHG is one of the main risk factors for several diseases such as cardiovascular diseases, stroke, retinopathy, renal failure, and neurologic complications (11-13).

The enzyme α-glucosidase is present in the brush border of the small intestine and catalyses the final step in the digestive process of carbohydrates to release absorbable monosaccharides resulting in increased blood glucose levels. Inhibition of this enzyme regulates the liberation of D-glucose from the dietary complex carbohydrates (14). The modern antidiabetic drugs are available in both oral and injectable forms. Among them, α-glucosidase inhibitors (AGIs) are among the main therapeutic agents for the treatment of type II diabetes. In diabetic patients, AGIs help to retard the metabolism of carbohydrates and regulate the elevation of high postprandial blood glucose level. Acarbose, voglibose, and miglitol are some of the currently available AGI drugs (15). Although AGIs prevent the hypoglycemic condition and also control the micro- and macro-vascular complications, side effects such as gastrointestinal irritation, flatulence, diarrhea, and abdominal discomfort occur with them (16).

Marine environment covers almost 70% of the earthꞌs surface and contains different biodiversity ecosystems (17). The microbial diversity of the marine environment is not entirely explored and organisms present in this environment are extremely rich sources of bioactive compounds (18, 19). Among them, actinobacteria, which are gram positive, filamentous bacteria with a high G+C ratio, are ubiquitous and potential organisms in the production of bioactive compounds (20). Amongst these actinomycetes, the genus *Streptomyces *is a potent producer of functional, bio-effective metabolites with wide pharmaceutical range having antimicrobial, anthelmintic, anti-tumor, and antiviral activities (21-23).The characteristics of marine actinobacteria are completely different from terrestrial actinobacteria and therefore, the bioactive compounds from marine actinobacteria are unique in their structure and properties (24). Even these marine actinobacteria are also widely distributed in association with biological sources such as fishes, molluscs, sponges, seaweeds, mangroves, besides seawater, and sediments (25). Nevertheless, the distribution of actinobacteria in the oceans is mostly unexplored and the existence of native marine actinobacteria in the marine environment remains scanty. Hence, the current work was undertaken to identify a unique compound from marine actinobacteria and to evaluate its postprandial anti-hyperglycemic activity.

## Experimental

*Chemicals*

The microbiological media such as Actinomycetes isolation agar (AIA), Kuster’s agar, Ken knights agar (KKA), starch casein nitrate agar (SCNA) and starch casein agar were purchased from HiMedia Laboratories, India. The p-nitrophenyl-a-D-glucopyranoside (PNPG), yeast aglucosidase (EC 3.2.1.20), sodium phosphate salts, sodium carbonate, and other solvents were purchased from Sisco Research Laboratories Pvt. Ltd. (SRL) - India. Streptozotocin and rat-intestinal acetone powder was obtained from Sigma-Aldrich, (USA).

*Isolation of actinobacteria*

The soil samples were collected from salterns, a coastal area in Kothapatnam, Ongole, Andhra Pradesh (15°30′ 0″ N, 80°3′ 0″ E), India. The soil samples were collected at the depth of 10-25 cm from the top layer of the salt pan and transferred into sterile polyethylene bags and transported to the Molecular and Microbiology Research Laboratory, Environmental Biotechnology Division, School of Bio Sciences and Technology, VIT University and were stored at 4 °C for further studies. Isolation and enumeration of actinobacteria were performed on selective media such as Actinomycetes isolation agar (AIA), Kuster’s agar, Ken knights agar (KKA), starch casein nitrate agar (SCNA), and starch casein agar. The soil samples were serially diluted using sea water up to 10^-7 ^dilution and one millilitre of the serially diluted samples were inoculated into different plates. All these media were supplemented with nalidixic acid and cyclohexamide (100 µg/mL) to avoid bacterial and fungal contamination. The plates were incubated at 28 °C and monitored periodically for 3 months for actinobacteria growth. Morphologically, distinct colonies were separated, purified, and maintained on actinomycetes isolation agar plates (26).

**Table 1:** Functional peaks of the isolated bioactive compound

**Wave No.**	**Correspondence Peaks**
2918.73, 2850.27	CH group
1736.58	C=O
1627.63	O-H (asymmetrical)
1420.32 – 1060.66	Pyrrole ring
764.637	C-Cl

**Figure 1:**
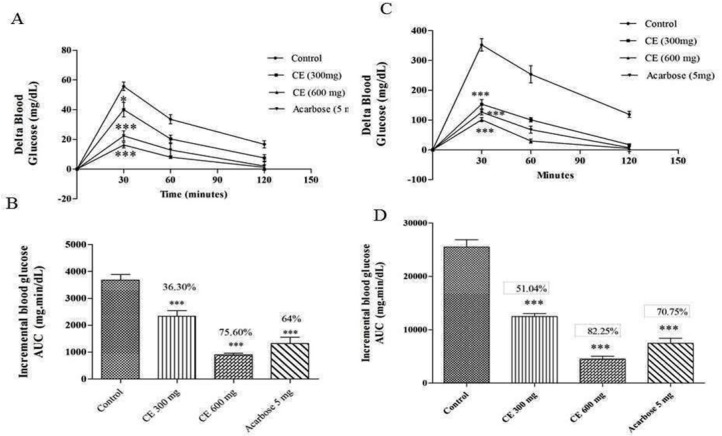
(A) and (C) curves showing the glycemic response in normal and diabetic rats after maltose loading along with CE. (B) and (D): incremental AUC_0-120 min _in diabetic and normal rats after maltose administration. Data are expressed as the mean ± SE (n = 6). *denotes *P* < 0.05 compared with control and ***-denotes *P* < 0.001 compared with control

**Figure 2:**
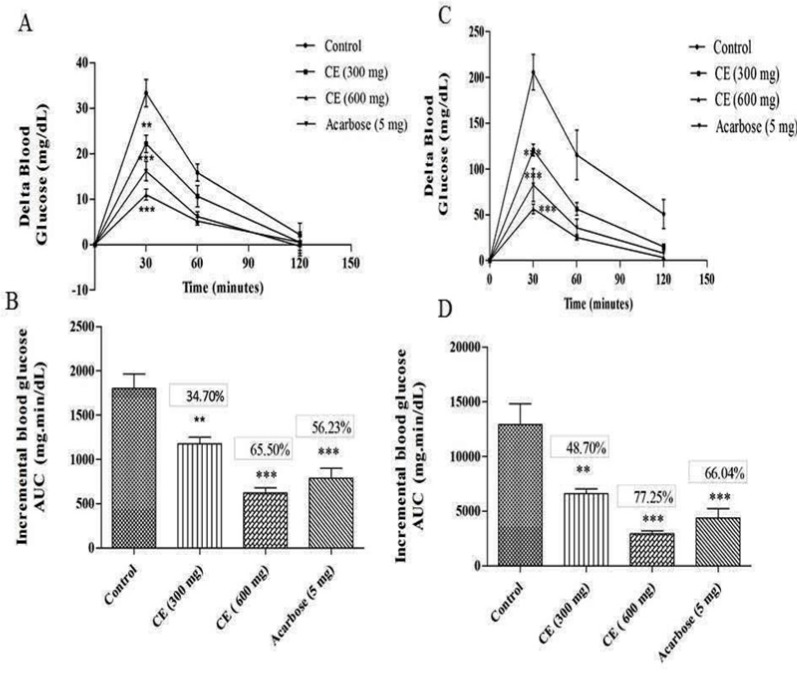
A) and (C) curves showing the glycemic response in normal and diabetic rats after sucrose loading along with CE. (B) and (D): incremental AUC_0-120 min _in diabetic and normal rats after sucrose administration. Data are expressed as the mean ± SE (n = 6). **denotes *P *< 0.01 compared with control and ***-denotes *P *< 0.001 compared with control

**Figure 3:**
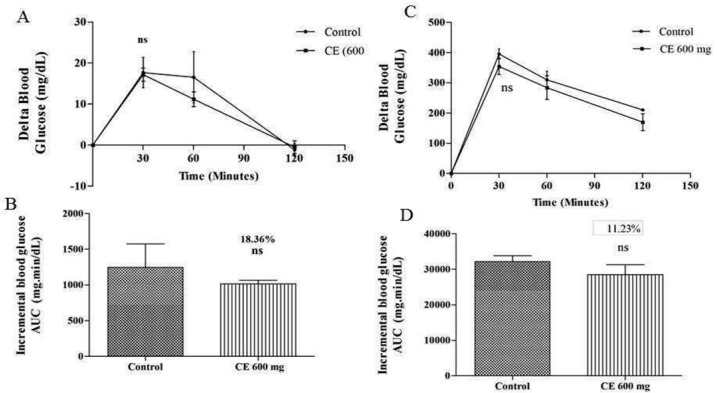
A) and (C) curves showing the glycemic response in normal and diabetic rats after glucose loading along with CE. (B) and (D): incremental AUC_0-120 min _in diabetic and normal rats after glucose administration. Data are expressed as the mean ± SE (n = 6). ns: not significant

**Figure 4.**
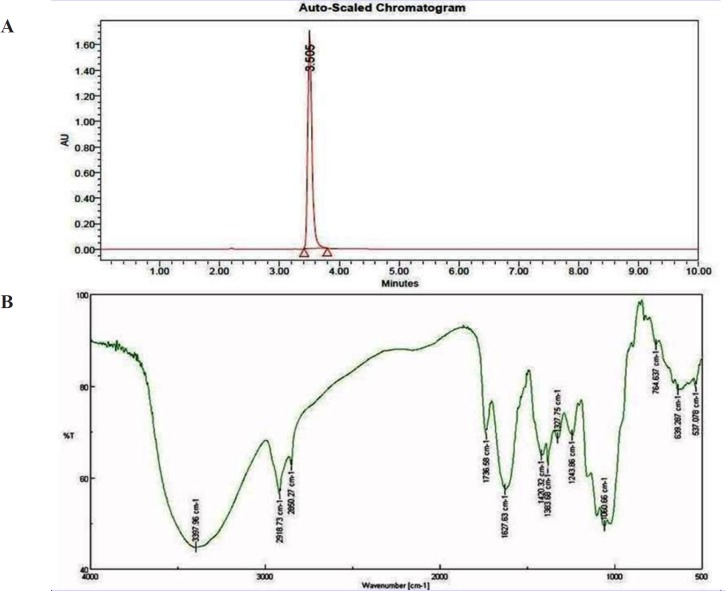
(A) HPLC chromatogram of isolated bioactive compound from *S. Coelicoflavus *SRBVIT13. (B) FTIR spectrum of bioactive compound isolated from *S. coelicoflavus *SRBVIT13.

**Figure 5:**
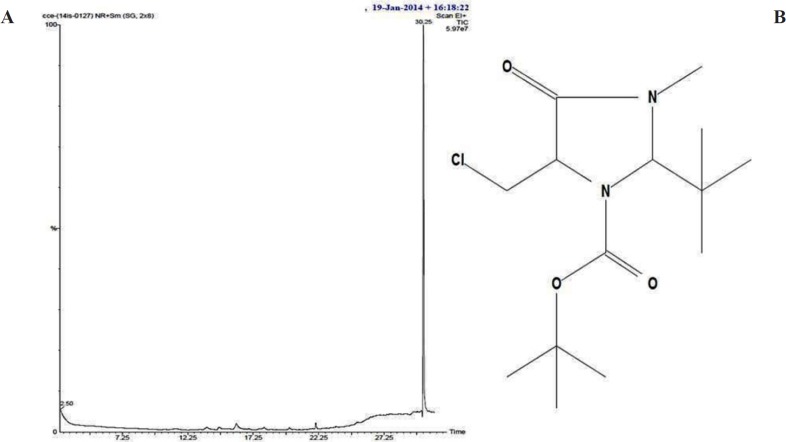
A), gas chromatographic spectrum of isolated bioactive compound from *S. coelicoflavus* SRBVIT13. (B), structure of proposed compound from *S. coelicoflavus* SRBVIT13

**Figure 6:**
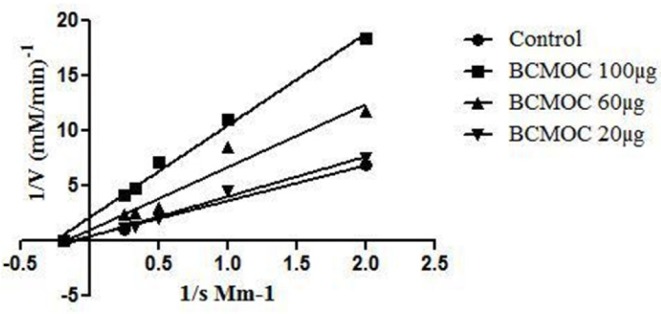
Enzyme Kinetics of α–glucosidase enzyme inhibitor by *S. coelicoflavus* SRBVIT13

*Fermentation process*

The purified actinobacterial isolates were inoculated into 50 mL of production medium (SS medium) in 100 mL Erlenmeyer flasks. The production medium was prepared with 50% marine water and 50% distilled water (pH 8.5) and incubated at 28 °C for 7 days on a rotary shaker at 10,000 rpm. After fermentation, the medium was harvested and centrifuged to remove cell debris. Then, collected supernatant was lyophilized and stored at 4 °C for further studies.

*Primary screening of actinobacteria for α-glucosidase inhibitor activity*

The actinaobacteria were primarily screened for their α-glucosidase inhibitory activity using slightly modified protocols of Brand-Williams *et al.*, (1995) and Jo *et al.*, (2009) for yeast and mammalian α-glucosidase enzyme inhibitory activity, respectively (27, 28). Briefly, 10 μL yeast α-glucosidase and rat α-glucosidase (1 U/mL in 0.1 M potassium phosphate buffer) were added separately to 50 μL of phosphate buffer (50 mM; pH 6.8) and mixed with 20 μL of varying concentrations of actinobacterial crude extract (20, 40, 60, 80 and 100 μg/mL) separately in a 96-well microtiter plate. The test sample mixture was pre-incubated for 5 min at 37 °C and 20 μL of 1 mM p-nitrophenyl α-D-glucopyranoside (PNPG) was added to the mixture as substrate. After incubation (37° C for 30 min), 50 µL of 0.1M sodium carbonate solution was added to terminate the reaction. Acarbose was used as positive control and distilled water as a negative control. The enzyme inhibition activity was estimated by recording the absorbance at 405 nm in a microtiter plate reader (Bio-TEK, USA). The percentage of inhibition was calculated as % Inhibition = [(AC - AS)/AC] × 100, where AC and AS are the absorbance of the control and test, correspondingly.

*Secondary screening of potent actinobacteria for Mammalian α-glucosidase inhibitor activity*

Three potent actinobacterial isolates were inoculated into 50 mL of production media (SS broth) in 100 mL Erlenmeyer flasks separately and incubated at 28 °C for 7 days on a rotary shaker (REMI) at 100 rpm. After incubation, the production media was harvested and centrifuged at 10,000 rpm for 10 min in a cooling centrifuge (REMI). The supernatant was collected and treated with different solvents like petroleum ether, ethyl acetate, n-butanol, chloroform, and hexane in the ratio of 1:1 in a separating funnel. The solvent layer was separated from the supernatant solution, the collected solvent samples were evaporated using a rotary vacuum evaporator (IKA, Germany). For mammalian α-glucosidase inhibitor assay, rat-intestinal acetone powder (200 mg) was dissolved in 4 mL of 50 mM ice cold phosphate buffer and sonicated for 15 min at 4 °C. After vigorous vortexing for 20 min, the suspension was centrifuged (10,000×g, 4 °C, 30 min) and the resulting supernatant was used for the assay. The condensed solvent crude extracts were further screened for mammalian α-glucosidase inhibitor activity.

*Evaluation of in-vivo postprandial hyperglycemic activity*

*Experimental animals*

Adult male albino Wistar rats were selected for the *in-vivo* study and they were kept in the animal house at Center for Biomedical Research, VIT University, Vellore, India. Animals weighing 160-210 g were kept in polycarbonate cage and maintained in a room under 12-h light/12-h dark cycle at 25 ± 2 °C and fed with standard rodent diet and water. All the experimental handling protocols were approved by the ethical committee in accordance with the Institutional Animal Ethics Committee, 1333/C/10/CPCSEA.

*Induction of diabetes*

The adult male albino Wistar rats were fasted for a period of 16 h. After fasting, the animals were induced for diabetes using streptozotocin (STZ). STZ was prepared in the fresh citrate buffer (0.1 M, pH 4.5) and administered intraperitoneally at the concentration of 45 mg/kg body weight. After three days of induction, the fasting blood glucose level of more than 250 mg/dL was considered as diabetic.

*Antihyperglycemic activity of S. coelicoflavus SRBVIT13 extract in maltose, sucrose loaded normal and diabetic rats*

A total of 24 normal rats were divided into four groups for assessing the antihyperglycemic activity of *S. coelicoflavus* SRBVIT13 in maltose loaded normal rats. Group I received maltose (2 g/kg body weight) alone and considered as control. Group II was treated with maltose (2 g/kg) and acarbose (5 mg/kg). Group III was co-administrated with maltose (2 g/kg) and chloroform extract of *S. coelicoflavus* SRBVIT13 (300 mg/kg) and group IV was treated with maltose (2 g/kg) and double the concentration of chloroform extract of* S. coelicoflavus* SRBVIT13 (600 mg/kg). After maltose administration in animals, the blood glucose level was monitored on 0^th^, 30^th^, 60^th^ and the 120^th^ min. of the experiment using the glucometer (Model: One touch Horizon™). The variation in blood glucose level after the oral maltose administration was measured and denoted as delta blood glucose. The same experimental animal model was followed for sucrose loaded diabetic rats (29).

*Antihyperglycemic activity of S. coelicoflavus SRBVIT13 in glucose loaded normal and diabetic rats*

To assess the antihyperglycemic activity of *S. coelicoflavus* SRBVIT13 by the inhibition α-glucosidase enzyme, a total of 12 normal rats were used and divided into two groups. The first group was considered as controls and received glucose (2 g/kg body weight), while the second group received glucose (2 g/kg) and chloroform extract of *S. coelicoflavus* SRBVIT13 (600 mg/kg). After treatment, the blood glucose level was measured on 0^th^, 30^th^, 60^th^, and the 120^th^ min of the treatment. The change in blood glucose level at the initial stage after glucose treatment was analysed and denoted as delta blood glucose (29).

*Isolation, purification and characterisation of bioactive compound from the chloroform extract of S. coelicoflavus SRBVIT13*

*Isolation of Bioactive compound*

The chloroform extract of *S. coelicoflavus* SRBVIT13 was further screened for purification of bioactive compound using preparative high performance liquid chromatography. In this experiment, Acetonitrile:Water (70:30) was used as a mobile phase for the separation of bioactive compound from chloroform crude extract. Different fractions were collected using preparative HPLC and the collected fractions were further tested for their mammalian α-glucosidase inhibitor activity. The fractions which were showing potent inhibition against mammalian α-glucosidase enzyme were further tested to identify the potent compound.

*Structural Elucidation of the lead compound*

FT-IR spectra were recorded on a FTIR spectrophotometer (Thermo Nicolet – Avatar-330, USA) in order to characterize the presence of functional groups in isolated bioactive compound. Two mg of the isolated pure compound was added to 200 mg of potassium bromide (KBr- FT-IR grade) and prepared as disc pellet. All the measurements were carried out in the range of 400-4000 cm^-1^ at a resolution of 4.0 cm^-1^. The isolated bioactive was further characterized using GCMS (GC: Clarus 680 and MS: Clarus 600 - EI) with Elite – 5MS column and the Helium gas (99.99%) was employed as a carrier gas under constant current rate of 1 cc/min. In this experiment, 1 µL of the sample was injected in the ratio of 10:1 and mass scan was processed under 50 to 600 Da. During the process, the oven temperature was designed from 60 to 300 °C. The NMR characterization of the bioactive compound was processed with the help of NMR spectroscopy (Ascend^TM^400, Bruker, Germany). The isolated bioactive compound was dissolved in deuterated chloroform and scanned for ^1^H-NMR, and ^13^C-NMR at 400 MHz in an NMR spectroscopy.

*Statistical analysis*

Statistical analysis was performed using one-way analysis of variance (ANOVA) followed by Dunnett’s Multiple Comparison Test using GraphPad Prism software. *P*-values of less than 0.05 were considered statistically significant. The delta blood glucose levels were expressed as mean ± SE for six animals in each group.

## Results

A total of 129 actinobacteria were isolated from the collected marine salt pan soils on different media. Among them, 47 morphologically distinct and pigmented isolates were selected, purified, and maintained in glycerol by storing at -20 °C until further use. The results reveal that the saltpan soils are rich in actinobacterial diversity.

*Primary and secondary screening of marine actinobacteria for α-glucosidase inhibitory activity*

Among the 47 isolates tested, only 11 isolates possessed α-glucosidase inhibitory activity against yeast α-glucosidase enzyme. Among them, three isolates, SRBVIT1, SRBVIT2 and SRBVIT3, with prominent activity were selected for further studies. In primary screening, yeast α-glucosidase inhibitory activity of SRBVIT1 showed 94.61% enzyme inhibition, whereas SRBVIT2 and SRBVIT3 showed 85.79 and 82.34% enzyme inhibition, respectively.

Bioactive compounds of various biological sources have the ability to inhibit yeast α-glucosidase enzyme that might lack the ability to inhibit mammalian α-glucosidase enzyme due to their variation in structural and functional properties. Rat α-glucosidase enzyme is most commonly used for testing α-glucosidase enzyme inhibitor studies, due to its 71.2% of similarity with human α-glucosidase enzyme (30). Hence, the selected three isolates were examined for their efficiency in inhibiting the mammalian α-glucosidase enzyme in addition to yeast α-glucosidase. The results revealed that, actinobacterial isolate SRBVIT1 showed significant inhibition (91.27%) on mammalian α-glucosidase activity followed by SRBVIT2 (84.12%) and SRBVIT3 (79.63%).

*Identification of potential isolates using 16S rRNA gene sequencing analysis*

Based on the results of primary and secondary screening of α-glucosidase inhibitor studies, the potent isolate, SRBVIT1, was selected for molecular characterization and it was identified as *Streptomyces coelicoflavus* using 16S rRNA gene sequencing analysis. In the BLAST search, 16S rRNA gene sequence of SRBVIT1 showed 98% of similarity with *Streptomyces coelicoflavus*. Founded on the BLAST search, the isolate SRBVIT1 was identified and named as Streptomyces coelicoflavus SRBVIT13. The gene sequence was later submitted in NCBI under the accession number of KF719279.

*Assessment of α-glucosidase inhibitory activity of different solvent extracts*

For the extraction of α-glucosidase inhibitor from the actinobacterial supernatant, different solvents *viz*., petroleum ether, ethyl acetate, n-butanol, chloroform, and hexane were used. Among these extracts, n-butanol and chloroform extracts of *S. coelicoflavus* SRBVIT13 showed significant inhibition of mammalian α-glucosidase enzyme activity. Specifically, chloroform extract of *S. coelicoflavus* SRBVIT13 showed 96.73% of enzyme inhibition, whereas the n-butanol extract showed 74.39% inhibition. The chloroform extract of *S. coelicoflavus* SRBVIT13 showed maximum inhibitory activity on mammalian α-glucosidase with an IC_50_ value of 41.24 μg/mL. Hence, the chloroform extract was used in *in-vivo* postprandial anti-hyperglycemic experiment.

Based on the results obtained in *in-vitro* evaluations, *S. coelicoflavus* SRBVIT13 chloroform extract was used in *in-vivo *experiments with male albino Wister rats. In animal models, diabetes was induced using STZ. The effect of chloroform extract in regulating blood glucose levels in both normal and diabetic rats after maltose or sucrose loading was measured.

*Antihyperglycemic effect of S. coelicoflavus SRBVIT13 extract in maltose and sucrose loaded normal and diabetic rats*

The chloroform extract of *S. coelicoflavus* SRBVIT13 was further evaluated for its anti-hyperglycemic activity in maltose loaded normal albino Wistar rats. Postprandial blood glucose level was determined after maltose loading to normal animals treated with chloroform extract and acarbose. After maltose loading in the control group, the 30 min average delta blood glucose level was increased up to 55.66 mg/dL. In test group, the animals were treated with chloroform extract along with maltose, the 30 min average delta blood glucose level was increased up to 40 mg/dL. This result reveals that the chloroform extract of *S. coelicoflavus* SRBVIT13 has the ability to control the elevation of maltose associated postprandial blood glucose level. Normal rats treated with chloroform extract showed 36.30% and 75.60% of blood glucose reduction in the doses of 300 and 600 mg/kg, respectively. Similar experiment was held out in diabetic animals. In this experiment, after maltose loading in diabetic control group, the 30 min average blood glucose level was increased up to 352.16 mg/dL. In the test groups, after maltose loading along with the chloroform extract at the doses of 300 and 600 mg/kg, the elevation of the delta blood glucose levels were regulated up to 153.14 mg/dL and 101.22 mg/dL, respectively. The test groups showed significant reduction of blood glucose level when compared to the animal group treated with maltose along with standard drug acarbose (Figure 1).

The results of the blood glucose regulation in sucrose loaded normal Wister rats showed that there was a significant reduction in 30 min average delta blood glucose level of the test group in two different doses (22.16 mg/dL and 11 mg/dL for of 300 and 600 mg/kg, respectively) compared to the control group (33.19 mg/dL). Likewise, a similar pattern was observed in diabetic test and control groups. The 30 min average postprandial blood glucose level of the diabetic control group was increased up to 205.05 mg/dL, while in test groups the delta blood glucose level was increased up to 120.66 mg/dL and 56.52 mg/dL with chloroform extract at the dose of 300 and 600 mg/kg, respectively. These results strongly confirm the ability of chloroform extract to regulate the sucrose mediated postprandial blood glucose level (Figure 2).

*Antihyperglycemic effects of S. coelicoflavus SRBVIT13 in glucose loaded normal and diabetic rats*

The regulation of blood glucose level by chloroform extract might be due to inhibition of α- glucosidase enzyme which was further confirmed by measuring the postprandial blood glucose level after glucose loading in normal rats. The outcomes indicate that there is no substantial difference in blood glucose levels of the control group (17.83 mg/dL at 30 min) and the test group (17.5 mg/dL) that received chloroform extract of 600 mg/kg along with glucose. The same movement was also noted in diabetic animals. As shown in Figure 3, there is no significant difference in blood glucose levels of control (395.16 mg/dL at 30 min) and test groups (360.5 mg/dL) that received chloroform extract of 600 mg/kg along with glucose. Hence, the results obtained indicate that the observed postprandial blood glucose regulation is due to the inhibition of α-glucosidase enzyme.

*Characterization and identification of α-glucosidase inhibitor*

Bioactive compounds were extracted from the culture supernatant of *S. coelicoflavus* SRBVIT13 using chloroform. Later, various bioactive compounds of the chloroform extract were separated by preparative HPLC using Acetonitrile: Water (70:30) as mobile phase. Further, the bioactive compound was characterized and identified using UV-visible spectrophotometry, FT-IR, GC-MS and NMR analysis. The UV-visible spectrum of the isolated bioactive compound showed λ _max_ at 280 nm. The purified bioactive compound was pale yellow in nature and soluble in water, chloroform, and DMSO. The HPLC chromatogram of the bioactive compound shows the purity of the product (Figure 4A).

The FT-IR analysis of the bioactive compound was carried out to identify the presence of functional groups. The FT-IR spectrum of the isolated bioactive compound showed peaks at different wave numbers corresponding to different functional groups and these results were summarized in Table 1 and Figure 4B. The GC-MS spectrum of the isolated compound showed a single peak at the retention time of 30.25 min, and the development of the single peak denotes the purity of the isolated bioactive compound. The peak developed was mass fragmented and the resultant bioactive compound was compared with the NIST library database (Figure 5A). The compound was found to be similar with 2-t-butyl-5-chloromethyl-3-methyl-4-oxoimidazolidine-1-carboxylic acid, t-butyl ester and molecular weight of thebioactive compound is 304. The structural identification of the bioactive compound was studied using 1HNMR and 13CNMR. 1HNMR: δ: 1.26 (s, 9H), 1.28 (9H, s), 2.17 (3H, s), 3.7 (2H, s), 3.8 (2H, s), 5.58 (1H, s), 5.62 (1H, s), 7.26 (2H, s). 13CNMR: 27.78 (-CH3), 29.51 (-CH3 of t-butyl group), 30.95 (-CH3 of t-butyl ester group), 46.74 (-CH2), 55.13 (t-butyl C), 82.49 (t-butyl ester C), 84.42 (C5), 93.04 (C2), 181.77 (-C=O), 207.0 (-COO). Based on the GC-MS and NMR analysis, the isolated bioactive compound was identified and confirmed as 2-t-butyl-5-chloromethyl-3-methyl-4-oxoimidazolidine-1-carboxylic acid, t-butyl ester (Figure 5B).

*Mammalian α-glucosidase inhibitory activity and enzyme kinetics of the isolated bioactivecompound*

The purified compound from *S. coelicoflavus* SRBVIT13 was studied for its enzyme inhibitory activity against mammalian α-glucosidase enzyme. In this study, the isolated potent compound showed significant inhibitory effects on α-glucosidase enzyme with an IC_50_ value of 32.08 µg/mL. The mode of enzyme inhibition of isolated and identified compound on mammalian α-glucosidase activity was studied using LB plot. The double-reciprocal plot displayed competitive inhibition of the enzyme. The K_m_ value increased with increasing concentration of the compound, while the V_max_ remained unaltered. Founded on the kinetics, the isolated bioactive compound showed non-competitive mode of enzyme inhibition (Figure 6).

## Discussion

Postprandial hyperglycemia (PPHG) is specified as an inflated increase of blood sugar level following a meal. The chloroform extract of *S. coelicoflavus* SRBVIT13 showed significant control of blood glucose in both normal and diabetic rats treated with maltose and sucrose. Similarly, Ganesan *et al.*, (2011) reported that a marine *Streptomyces* sp. isolated from the marine sediment samples showed significant reduction of the postprandial blood glucose level in *in-vivo* condition (31). In diabetic animals, the regulation of postprandial blood glucose level was improper due to the lack of insulin secretion (32). Whereas, in the current study, in diabetic animals treated with maltose and sucrose, the chloroform extract of the bacteria controlled the elevation of postprandial blood glucose. The primary factor for evaluating the controlled blood glucose level in diabetic animals might be ascribable to the inhibitory action of chloroform extract on the disaccharide metabolism. The α-glucosidase inhibitor (AGI) drugs are currently one of the main groups of therapeutic agents in the treatment of type II diabetes. They can help to delay the metabolism of carbohydrates and regulates the elevation of postprandial blood glucose level in diabetic patients.

Microbiologically derived enzyme inhibitors are widely used in industries for various applications. However, enzyme inhibitors from marine microorganisms were less studied (33). The secondary metabolites from actinobacteria may act as enzyme inhibitors for several diseases (34, 35). Recently, Karthik *et al.*, (2014) reported that a peptide compound from *Streptomyces *sp., LK3, isolated from marine sediments, showed significant inhibition of protease enzyme and also showed anti plasmodial activity (36). Hence, the screening of enzyme inhibitors from marine actinobacteria will serve as platform for unique inhibitors.

In the current study, the results obtained reveals that the saltpan soils are rich in actinobacterial diversity. In a previous study, SivaKumar, (2001) documented a number of marine actinobacterial colonies on the M6 medium (37). Similarly, Karthik *et al.*, (2010) studied the selective enhancing potential of M6 media on actinobacteria isolation from Andaman Nicobar marine sediments, while the other media used were found less effective in the recovery of actinobacterial isolates (26). In another study, Baskaran *et al.*, (2011) reported that the starch casein agar and Kusterꞌs agar are more influential for the marine actinobacterial isolation from the soil samples (38). Our results are in agreement with the previous reports and, in particular, the media. Actinomycetes isolation agar and starch casein agar recovered more actinobacterial colonies compared to other media.

Carbohydrate based diet plays main role in increasing the elevation of postprandial blood glucose by the immediate absorption of complex sugars, as α-glucosidase converts disaccharide to the simplest form of sugars (39). Thus, the α-glucosidase inhibitor prevents the metabolism of disaccharides and controls the sudden release of high amounts of glucose in the blood stream (30).

In the present study, a total of 47 actinobacterial isolates were recovered from the salt pan soil samples, and each isolate was primarily screened *in-vitro* for yeast α-glucosidase inhibitory activity. Chloroform extract of *S. coelicoflavus *SRBVIT13 showed 96.73% enzyme inhibition, whereas the n-butanol extract showed 74.39% inhibition. *S. coelicoflavus *SRBVIT13 showed significant inhibitory activity against yeast and mammalian α-glucosidase when compared to the other two isolates. Hence, *S. coelicoflavus *SRBVIT13 was further tested in *in-vivo *condition. In a previous study, Suthindhiran *et al.*, (2009) reported that organic solvent extract of an actinomycete strain VITSDK3, isolated from marine sediment sample collected at Marakkanam, the southern coast of India, showed 74.43% inhibition of α-glucosidase activity and the strain was characterized using polyphasic taxonomy and identified as *Micromonospora *sp. (40). Likewise, Pratibha *et al.*, (2013) reported anti-diabetic activity of *Streptomyces *sp. VITPK9 isolated from brine springs located in Thoubal district, Manipur, India; and another strain *Streptomyces *sp. VITSTK7 isolated from marine sediments collected from Puducherry coast, Tamil Nadu, India. Among them, the ethyl acetate extract (500 μg/mL) of VITSTK7 showed significant inhibition (64.3%) of α-glucosidase activity compared to VITPK9. However, inhibition of α-glucosidase activity of the isolates used in this study exhibited more promising results with highest inhibitory activity compared to other reports of marine actinomyctes (41).

In *in-vivo *studies, the chloroform extract of *S. coelicoflavus *SRBVIT13 showed potent ability in controlling the postprandial blood glucose level. Normal rats are treated with chloroform extract along with maltose showed 36.30% and 75.60% of blood glucose reduction in the dosage of 300 and 600 mg/kg, respectively. Whereas, the diabetic rats showed 51.04% and 82.25% of blood glucose reduction in the same dosages, respectively (Figure 1). Similarly, the normal rats were treated with the chloroform extract along with sucrose showed 34.70% and 65.50% of blood glucose reduction in the dosage of 300 and 600 mg/kg, respectively. Whereas, the diabetic rats showed 48.70% and 7.25% of blood glucose reduction at the same dosages, respectively (Figure 2).

## Conclusion

Antihyperglycemic effects of the *S.coelicoflavus* SRBVIT13 have been revealed by in-vitro and animal studies. The identified bioactive compound may be considered as a potent α-glucosidase enzyme inhibitor and could be considered as a candidate for a therapeutic approach for the regulation of postprandial hyperglycemia after further clinical trials. 

## References

[1] Ramachandran A, Das AK, Joshi SR, Yajnik CS, Shah S, Prasanna Kumar KM (2012). Current status of diabetes in India and need for novel therapeutic agents. J. Assoc. Physicians India.

[2] Das SK, Elbein SC (2006). The genetic basis of type 2 diabetes. Cellscience.

[3] Chen L, Magliano DJ, Zimmet PZ (2012). The worldwide epidemiology of type 2 diabetes mellitus-present and future perspectives. Nat. Rev. Endocrinol.

[4] Stumvoll M, Goldstein BJ, Van Haeften T (2005). Type 2 diabetes: Principles of pathogenesis and therapy. Lancet.

[5] Zimmet P, Alberti KG, Shaw J (2001). Global and societal implications of the diabetes epidemic. Nature.

[6] Alberti KG (2001). Treating type 2 diabetes–today’s targets, tomorrow’s goals. Diabetes Obes. Metab.

[7] Dandona P, Aljada A, Bandyopadhyay A (2004). Inflammation: The link between insulin resistance, obesity and diabetes. Trends Immunol.

[8] Stumvoll M, Goldstein BJ, Van Haeften TW (2008). Type 2 diabetes: Pathogenesis and treatment. Lancet.

[9] Meyer C, Woerle HJ, Dostou JM, Welle SL, Gerich JE (2004). Abnormal renal, hepatic, and muscle glucose metabolism following glucose ingestion in type 2 diabetes. Am. J. Physiol. Endocrinol. Metab.

[10] Mitrakou A, Kelley D, Mokan M, Veneman T, Pangburn T, Reilly J, Gerich J (1992). Role of reduced suppression of glucose production and diminished early insulin release in impaired glucose tolerance. N. Engl. J. Med.

[11] Gallwitz B (2009). Implications of postprandial glucose and weight control in people with type 2 diabetes: Understanding and implementing the International Diabetes Federation guidelines. Diabetes Care.

[12] Gerich JE (2003). Clinical significance, pathogenesis, and management of postprandial hyperglycemia. Arch. Intern. Med.

[13] Lin HJ, Lee BC, Ho YL, Lin YH, Chen CY, Hsu HC, Lin MS, Chien KL, Chen MF (2009). Postprandial glucose improves the risk prediction of cardiovascular death beyond the metabolic syndrome in the non-diabetic population. Diabetes Care.

[14] Hillebrand I, Boehme K, Frank G, Fink H, Berchtold P (1979). The effects of the alpha-glucosidase inhibitor BAY g 5421 (acarbose) on meal-stimulated elevations of circulating glucose, insulin, and triglyceride levels in man. Res. Exp. Med.

[15] Van de Laar FA, Lucassen PL, Akkermans RP, van de Lisdonk EH, Rutten GE, Weel C (2005). Alpha-glucosidase inhibitors for patients with type 2 diabetes: Results from a Cochrane systematic review and meta-analysis. Diabetes Care.

[16] Heiner L (2002). Acarbose an update of its therapeutic use in diabetes treatment. Clin. Drug Investig.

[17] Valli S, Suvathi SS, Aysha OS, Nirmala P, Vinoth Kumar P, Reena A (2012). Antimicrobial potential of Actinomycetes species isolated from marine environment. Asian Pac. J. Trop. Biomed.

[18] Hong K, An HG, Qing YX, Hao G, Ling Z, Hai PL, Hai PY, Jia L, Xin SY, Michael G, Ji SR (2009). Actinomycetes for marine drug discovery isolated from mangrove soils and plants in China. Mar. Drugs.

[19] Solanki R, Khanna M, Lal R (2008). Bioactive compounds from marine actinomycetes. Indian J. Microbiol.

[20] Goodfellow M, Williams ST (1983). Ecology of actinomycetes. Annu. Rev. Microbiol.

[21] Berdy J (2005). Bioactive microbial metabolites. J. Antibiot.

[22] Karthik L, Kumar G, Keswani T, Bhattacharyya A, Reddy BP, Bhaskara Rao KV (2013). Marine actinobacterial mediated gold nanoparticles synthesis and their antimalarial activity. Nanomedicine.

[23] Reddy NG, Ramakrishna DPN, RajaGopal SV (2011). A morphological, physiological and biochemical studies of marine Streptomyces rochei (MTCC 10109) showing antagonistic activity against selective human pathogenic microorganisms. Asian J. Biol. Sci.

[24] Haefner B (2003). Drugs from the deep: Marine natural products as drug candidates. Drug Discov. Today.

[25] Dharmaraj S (2010). Marine Streptomyces as a novel source of bioactive substances. World J. Microbiol. Biotechnol.

[26] Karthik L, Gaurav K, Bhaskara Rao KV (2010). Diversity of marine actinomycetes from Nicobar marine sediments and its antifungal activity. Int. J. Pharm. Pharm. Sci.

[27] Brand-Williams W, Cuvelier M, Berset C (1995). Use of free radical method to evaluate antioxidant activity. Food Sci. Technol.

[28] Jo SH, Ka EH, Lee HS, Apostolidis E, Jang HD, Kwon YI (2009). Comparison of antioxidant potential and rat intestinal α-glucosidases inhibitory activities of quercetin, rutin, and isoquercetin. Int. J. Applied Res. Nat. Prod.

[29] Shihabudeen HMS, Hansi P, Kavitha T (2011). Cinnamon extract inhibits a-glucosidase activity and dampens postprandial glucose excursion in diabetic rats. Nutr. Metab.

[30] Casirola DM, Ferraris RP (2006). Alpha-Glucosidase inhibitors prevent diet induced increases in intestinal sugar transport in diabetic mice. Metabolism.

[31] Ganesan S, Raja S, Sampathkumar P, Sivakumar K, Thangaradjou T (2011). Isolation and screening of α-glucosidase enzyme inhibitor producing marine actinobacteria. Afr. J. Microbiol. Res.

[32] Pospisilik JA, Martin J, Doty T, Ehses JA, Pamir N, Lynn FC, Piteau S, Demuth HU, McIntosh CH, Pederson RA (2003). Dipeptidyl peptidase IV inhibitor treatment stimulates beta-cell survival and islet neogenesis in streptozotocin-induced diabetic rats. Diabetes.

[33] Imada C (2004). Enzyme inhibitors of marine microbial origin with pharmaceutical importance. Mar. Biotechnol.

[34] Martin JF, Demain AL (1980). Control of antibiotic biosynthesis. Microbiol. Rev.

[35] Imada C (2005). Enzyme inhibitors and other bioactive compounds from marine actinomycetes. Antonie Van Leeuwenhoek.

[36] Karthik L, Gaurav K, Keswani T, Bhattacharyya A, Chandar SS, Bhaskara Rao KV (2014). Protease inhibitors from Marine actinobacteria as a potential source for antimalarial compound. PLoS One.

[37] SivaKumar K (2001). Actinomycetes of an Indian Mangrove (Pitchavaram) environment: An inventory [dissertation]. Annamalai University, India.

[38] Baskaran R, Vijayakumar R, Mohan PM (2011). Enrichment method for the isolation of bioactive actinomycetes from mangrove sediments of Andaman Islands, India. Malays. J. Microbiol.

[39] Dahlqvist A, Borgstrom B (1961). Digestion and absorption of disaccharides in man. Biochem. J.

[40] Suthindhiran RK, Jayasri MA, Kannabiran K (2009). α-glucosidase and α-amylase inhibitory activity of Micromonosporasp VITSDK3 (EU551238). Int. J. Integr. Biol.

[41] Pratibha S, Thenmozhi M, Kannabiran K (2013). Screening of glycolytic enzyme inhibitory activity of Streptomyces isolates from brine spring and marine sediments of India. Int. J. Pharm. Sci. Rev. Res.

